# Book Reviews

**Published:** 1825-05

**Authors:** 


### CRITICAL ANALYSIS

OF

### ENGLISH AND FOREIGN LITERATURE,

RELATIVE TO THE VARIOUS BRANCHES OF

### Medical Science.

Quæ laudanda forent, et quæ culpanda, vicissim

Illa, prius, cretâ; mox hæc, carbone, *notamus*.—PERSIUS.

### DIVISION I.

### ENGLISH.

ART. I.—*An Essay on Venereal Diseases, and the Uses and Abuses of Mercury in their Treatment. Illustrated by Drawings of the different Forms of Venereal Eruptions.* Second Edition. By RICHARD CARMICHAEL, M.R.I.A. Vice-President of the Royal College of Surgeons in Ireland, and one of the Surgeons of the Richmond Surgical Hospital, Dublin, &c. &c. &c.—8vo. pp. 376. Longman and Co. London, 1825.

WE are aware of the nausea and distaste with which the majority of our readers may view the title of the present article,—feelings not dissimilar to those produced by the sight and smell of the very best dinner upon the olfactory and optic nerves of a man who has just risen from the enjoyment of that meal; for such, indeed, has been the overflowing supply of publications, of all descriptions, upon the subject of Syphilis of late years, that the greediest devourer of novelties must, we think, be more than satisfied. Nevertheless, Mr. CARMICHAEL's opinions deserve our attention, and command our notice; and, as they have not come under our cognizance since we commenced our literary labours, we have determined to discharge our consciences at once, and have done, we trust for some time, with this very hackneyed subject. In saying this, we would by no means wish it to be thought that we consider the inquiry as either unimportant or uninteresting; but we are free to confess that, with the present stock of facts which we possess, it is scarcely possible for the most ingenious writer to hit upon any thing having the charm of novelty, or leading to any new or important change in practice.

Among the various and conflicting opinions that have from age to age been broached upon the subject of syphilis, two only appear to merit much notice in these days. One of these views consists in the belief that the venereal disease is, like the late French republic, one and indivisible; the other sect adhering to the persuasion that there are several venereal diseases, but that one only deserves, *par excellence*, to be called syphilis, being attended with a peculiar train of symptoms, and being the result of a particular description of ulceration. Of this latter persuasion is the author before us; and we shall presently proceed to examine in what manner he supports this opinion,—taking, however, this opportunity of remarking, that we conceive he has entirely overthrown his own hypothesis, by the confession that the true syphilis is like the other forms of venereal ulceration and disease curable without mercury; which negation was, in fact, the strong hold of the first edition of his work.

The contests that have arisen as to the source from whence this disease originated, form another object of inquiry, but which we do not intend to enter into deeply: it is only necessary to premise, that it has been contended by some to have existed from the most remote antiquity; whilst, among those who advocate its more modern origin, there is great dissention as to whether it was imported to Europe from Africa or America. This part of the discussion we do not profess to enter into at all: nor, indeed, should we deem the former part of the inquiry of much importance, but as connected with the point to be determined,—viz. that of a multiplicity of venereal poisons.

Of Mr. Carmichael's work we may observe generally, that it consists of seven chapters,—is dedicated, in very handsome terms, to Sir James Macgrigor,—and is illustrated with five plates, the execution of which, however, we cannot praise. The style of Mr. Carmichael's work is perspicuous; and we must, above all, bear in mind, that the meed of early inquiry is eminently his due, since the first edition of his labours on this subject was published as early as the year 1814. We now proceed to discuss the work *seriatim*.

The first chapter contains "Observations on those Morbid Poisons which stand in nearest relation to the Syphilitic, and Evidence of the Existence of Venereal Diseases which do not arise from that Poison." The first pages of this chapter are devoted to the consideration of the Yaws, the Sivvens, and a disease somewhat similar, which is found in certain districts of Canada, in the maritime provinces of Norway, and some parts of Sweden and Russia. Into this discussion we shall not enter, as it does not immediately concern the subject in hand, and as most of the authorities quoted by our author are well known, and have often been before the profession. We pass on, then, to the first argument adduced by our author, which is ushered in by the following sentence:—

"The organs of generation are subject to a variety of ulcers, destitute of the characteristics of chancre—the hardened edge and base; yet most practitioners look on them as chancres, and treat them as such, imagining that inflammation, peculiarity of constitution, or some accidental circumstance, has deprived them of the usual character of the primary syphilitic ulcer." (P. 17.)

To discover whether chancres may be altered by peculiarity of constitution or accidental causes, continues our author, let us look at the analogous symptoms of other contagious diseases; and, as he finds that neither the pustule of the small-pox, nor the vesicle of the cow-pock, are so changed, he draws the inference that the syphilitic poison must also pursue one and the same course. But surely this analogy cannot hold good respecting the venereal poison; and our author himself, in the first paragraph of his work, virtually admits that there is no analogy between these diseases; for he says, "It is a curious fact that morbid poisons which excite considerable fever, such as the small-pox and measles, yield to the powers of the constitution, and are capable of a spontaneous cure. The increased action of the system is sufficient to overcome the poison; but in syphilis it seems to be nearly, if not altogether, insufficient: thence, it would appear, arises the necessity, in this disorder, of artificially raising an action by means of mercury, which, though capable of superseding the influence of the poison, does not, however, extinguish (like the natural fever of the small-pox) the susceptibility of receiving the disorder again." (P. 1, 2.) Here then, by his own confession, are two striking distinctions between the known laws of these poisons; and further on, when discussing the symptoms of the papular venereal disease, he affirms that this train of symptoms is the result of *two* different kinds of sores, as well as of gonorrhœa virulenta; added to which, is it really true that the constitution affords no modification or change in either the small-pox or the vaccine pustule? We believe the exact contrary to be the fact; and we cannot for a moment doubt that, in parts of such different structure as the organs of generation, and under the various circumstances of diet, intemperance, or distempered habit of body, what in one man will be a simple sore, in another may assume that peculiar character, which, since the publication of Mr. HUNTER's work on the Venereal Disease, has been by some held to be an indispensable character of chancre.

Mr. Carmichael next proceeds to notice the frequency of ulcers of the genitals, not arising from the poison of syphilis, proved by the testimony of authors long antecedent to the supposed date of that disease; and of these he gives us many apt quotations, beginning with Hippocrates, Galen, and Celsus, and ending at the period of the invasion of the venereal disease. To these we could add several other testimonies, which he has either overlooked or passed by as unnecessary to prove what no one, who is acquainted with the ancient authors, can deny; and what, indeed, might *a priori* have been anticipated by any unlearned man: for why should the penis and the female genital organs,—parts so exquisitely organised, and so largely supplied with blood and with mucous glands,—be exempt from breach of surface, and consequent ulceration, more than any other part of the human body? But we shall be contented with inserting two quotations only, to show that we give full credit to the antiquity of ulcers on the organs of generation. The first quotation is from PLINY,* who says, "Navegabam per Larium nostrum, cum Senior Amicus ostendit mihi villam, atque etiam cubiculum, quod in lacum prominet. Ex hoc (inquit) aliquando municeps nostra cum marito se precipitavit causam requisivi. Maritus ex diutino morbo *circa velanda corporis ulcerebus putrescebat.* Uxor ut inspiceret, exigit, neque enim quinquam fidelius judicaturum, possetne sanari. Videt, desperavit, hortata est ut moreretur, comes que ipsa mortis, dux, immo et exemplum et necessitas fuit. Nam se cum marito ligavit, adjecitque in lacum." The good Bishop PALLADIUS, who is our second instance, informs us of a man, who, in consequence of connexion with a female, was affected with a gangrene of the penis, which he attributes to divine wrath, and in consequence of which the sinful member fell off at the end of six months. We could accumulate these stories almost *ad infinitum;* but, since all the above writers are silent as to any constitutional consequences proceeding from these ulcerations, and moreover as neither the satirists nor poets of those times have any allusion to after consequences, we must infer, from their very silence, that none were to be met with; whilst the sudden inundation of writings on the new scourge which appeared towards the close of the fifteenth century,—the great terror it inspired,—its horrible after-consequences, so loudly and absolutely insisted upon,—the spreading of this disease among sovereigns, cardinals, nobles of both sexes, sufficiently prove that there was a something new,—something which the diseases of the genitals heretofore had not been accustomed to produce. Nay, so rapid was the progress of the new disease, that the island of Inch Keith was appointed as the place of banishment for the syphilitic patients of Scotland, as early as the year 1495; and we find in ASTRUC a copy of the arrêt, published in Paris in the year 1498, ordering all those affected with the *grosse verole* to quit Paris by the gate of St. Dennis and St. Jaques, when they were to be met by proper persons, to supply them, if strangers, with a certain sum to take them to their homes; and a multitude of other regulations, which it is not necessary for us to repeat. Now, though we are willing to admit that all these precautions

* Lib. 6. Epis. 25.

prove a total ignorance of the real nature of the complaint, and which is further evinced by the opinions entertained by many of the earlier writers, that this new affliction proceeded from the malignant influence of the planets, or from some undefined occult quality of the air; but it is too much to believe, with our author, that, although the writers prior to the fifteenth century do not notice constitutional symptoms as consequent upon ulcerations of the genitals, yet "they (the ancients) might have had these symptoms before their eyes every day, although they might not have had any suspicion of their origin, or a conception of the connexion that exists between the primary ulcer of a morbid poison and the constitutional maladies that follow it." (P. 26.) This we cannot but think a very unfounded imputation upon the sagacity of our ancestors: indeed, too much to expect us readily to believe, merely for the sake of supporting a theory.

But we are still more surprised at Mr. Carmichael's attempt to support his argument by the authority of Mr. BECKET's papers in the Philosophical Transactions. We had thought that writer's merits had been long since set at rest; for, surely, any one who dispassionately reads the authorities he quotes cannot fail to be astonished at the very forced construction he has put upon one or two rather equivocal expressions; and we venture to say, that whoever will take the pains to read the chapter which Astruc* has devoted to the consideration of Becket's authorities, will arise from it with a perfect conviction that it is most triumphant and unanswerable. However, as Astruc may be suspected of an undue bias, we shall venture to extract a paragraph from an author of undoubted learning, great research, and totally free from all preconception or prejudice on the subject. This author, after summing up all the evidence adduced from the authorities quoted by Becket and others, concludes thus—"However, I shall enlarge no further, as Mr. Le Clerc rightly observes, if this distemper had been ancient, it must have been taken notice of, if not by the practising physicians, by the poets at least. So I think 'tis a very good argument that it was not at all known in the time of the earliest of these writers; otherwise so fruitful a subject would never have escaped the raillery of Dante, Petrarch, and Boccace."†

Our narrow limits have obliged us to contract very much what we could further have urged upon this point. We shall not, however, be afraid to recur to it, should it be necessary, on any future occasion. We conceive that the above considerations will be sufficient to establish the position, that, although ulcers

* Lib. 1, chap. 7.

† FRIEND's History of Physic, vol. ii.

of the genitals of various kinds were occasionally met with from the most remote antiquity, still it is evident that they were not followed by any constitutional after-consequences, and therefore bore no relation to the disease afterwards known by the name of *syphilis, mal de Francos, Neapolitan disease,* and other appellations, demonstrating that the nations of Europe looked upon it, not only as a new scourge, but as a reproach, which they were anxious to throw each upon his neighbour. In relation to this, we cannot but smile at the dexterity with which honest AMBROSE PARÉ throws this stigma from his countrymen. He observes, that many call this disease the French disease, others the Neapolitan: "For my part," he continues, "I believe it to be the French disease when a Frenchman is the subject of it, and a Neapolitan disease when it attacks a native of Naples."

We now come to another object of the inquiry, and this relates to the particular kind of sore to which Mr. HUNTER chose to limit the definition of a chancre. This he was induced to do, no doubt, because that description of sore was the most usual form of ulceration met with at the time he wrote his Treatise on the disease; but now that kind of sore has become so rare as to be almost extinct. Yet, because Mr. Hunter thought it right to exclude from the list of syphilis certain anomalous complaints that would not come within the range of his definition, that were mixed up with circumstances that could not satisfactorily be referred to the usual known history of the venereal disease, it is by no means a fair inference to suppose that all forms of ulceration, except a particular one, are the results of other poisons, and thus to convert the exception at once into the general rule. Let us recollect the difficulty of arriving at the *truth* in any history of a case of ulcers on the genitals; let us consider the many motives for deception; and we shall clear away no small number of those improbable and irreconcileable stories, with which the history of medicine and surgery is overwhelmed. In our own times, and in our own country, we find, in the revolution of a few years, one particular form of ulceration prevailing, and then as suddenly disappearing. In Portugal, for example, the phagedenic sore prevails occasionally to a frightful degree; whereas, during a residence of some months in one of the largest cities of Portugal, we scarcely saw a case of it. The same has been the case in Ireland. Is it not also frequently observed, that different men shall suffer very differently, both in the extent and severity of their disease, from connexion with the same woman? Is there not, therefore, in such instances, abundant proof that a different state of the constitution *does* contribute to produce a modification of the poison? and is it not both unphilosophical and dangerous to attribute that which may reasonably be referred to the operation of one cause, to a multiplicity of agents? We do not hesitate to affirm that the doctrine of pseudo-syphilis has been productive of manifold evils: it has led to the utmost vagueness and uncertainty in practice, —it has conduced to endless refinements,—and it has inundated both town and country with every form of secondary symptom, in a proportion that was never remembered by the oldest practitioner.

We think that our author has been particularly unhappy in the cases he has quoted from Mr. ABERNETHY, (page 39, 40;) since, in the first case, the gentleman's sores were *not* followed by constitutional symptoms, and, being always produced by connexion with the same woman (believed to be in health), we have certain proof that those sores must have been occasioned by some peculiar state of the habit. The second case of the apparent gonorrhœa, and consequent bubo, is really too trifling to be noticed seriously.

This long disquisition has led us to nearly the middle of Mr. Carmichael's second chapter; and we shall now pursue our analysis without further interruption or frequent comment. Having expressed our belief of there being but one venereal poison, and likewise endeavoured to establish the fact of its comparatively modern origin, we shall reserve what we have to say upon the late investigation of this subject, and the non-mercurial practice, to the end of this article.

The following conclusions, then, may be considered as the texts of our author's work, which are commented upon and explained in the succeeding chapters:—

"First. That the syphilitic chancre is attended by the scaly eruptions, lepra and psoriasis, an excavated ulcer of the tonsils, and pains and nodes of the bones.

"Second. That the simple ulcer, without induration, raised edges, or phagedenic surface,—gonorrhœa virulenta,—and excoriation of the glans and prepuce, are followed by a papular eruption, which ends in desquamation, pains in the joints resembling those of rheumatism, soreness of the fauces, and frequently swelling of the lymphatic glands of the neck; but that, in a vast number, not a single instance was observed in which nodes were an attendant upon this eruption.

"Third. That the ulcer with elevated edges, in the few instances in which I had an opportunity of tracing it to its constitutional symptoms, was followed by a pustular eruption, which terminated in mild ulcers, pains in the joints, and ulcers in the throat, but no appearance of nodes; yet that the instances in which I had an opportunity of witnessing distinctly the connexion between the primary and secondary symptoms of this poison, were too few to form a decided conclusion with respect to this particular.

"Fourth. That the phagedenic and sloughing ulcers are generally attended by constitutional symptoms of peculiar obstinacy and malignancy, viz. pustular spots and tubercles, which formed ulcers that spread in general with a phagedenic edge, and heal from the centre; extensive ulceration of the fauces, particularly of the back of the pharynx, obstinate pains of the knees and other joints, while nodes are frequently present, and the bones of the nose are occasionally affected.

"Fifth and last. That when an eruption, no matter what its character may be, is on a surface which is opposed by another, as on the fossa of the nates, upper part of the inside of the thighs, or in the axilla, the spots, if they do not ulcerate, extend into soft, moist elevations of the cutis, which ought to be treated according to the nature of the disease to which they belong. Thus, if they are syphilitic, with mercury; or if papular, pustular, or tubercular, with the remedies recommended for the specific disorder. According to the established practice, these condylomatous swellings, as they are called, are universally treated with mercury; but I have, in innumerable instances, cured them, and the other symptoms with which they are accompanied, without the exhibition of a particle of that medicine." (P. 53—55.)

This leads us to the consideration of Chapter III. on the *Papular Venereal Disease.* The class of venereal complaints liable to be attended with an eruption of papulæ is, says our author, the most simple, the most common, and most easily cured, of any of the forms of that disease: the primary symptoms are either a simple ulcer, without induration, elevated edges, and phagedena; or patchy excoriation of the glans or prepuce; or the gonorrhœa virulenta. This simple ulcer, though not often seen by the surgeon in its commencement, exhibits at first a small pustule, then forms a crust, which, falling off, leaves an ulcer of an oval or round shape, excavated, and with a surrounding redness. This Mr. C. calls the simple primary venereal ulcer. From three to six weeks may be mentioned as the average period of its continuance; but he observes, that the *appearance* and duration of this, as of every other primary sore, is liable to be modified by the state of the constitution, mode of living, exposure to various irritating causes, neglect and want of cleanliness, &c. These ulcers are more commonly found on the glans and internal prepuce, and they frequently excite phymosis; and therefore our author thinks it very probable that this is the identical ulcer mentioned by Celsus as inducing phymosis. But here we must be allowed to remark, that the formation of the prepuce has, we conceive, more to do with the production of phymosis than the nature of the sore. If a man has a long prepuce, any sore attended with much inflammatory action will induce phymosis; and this is one of the overstrained adaptations of ancient authority, which we so frequently have occasion to remark in the perusal of this volume.

In mentioning the patchy excoriation of the glans or internal prepuce, often attended with phymosis also, we find our author endeavouring to prove that this affection is identical with gonorrhœa; and he supports his opinion by the authorities of MUNRO and WHATELY, though we do not perceive with what justice. He says that both parts (the urethra and the internal surface of the prepuce) have the same continuity of surface, and that there exists a great similarity in the affections in question. From the frequent occurrence of the primary sore, excoriation, and gonorrhœa, existing together in the same patient, and from the fact that each occasions the same train of constitutional symptoms, we have, adds Mr. C., strong grounds for concluding that they arise from the same identical poison. Here argument becomes of no use, it is mere fact that can decide the question; and we do not hesitate to say, that the junction of these three forms of disease is by no means so common or universal as to justify such a general inference. We likewise disbelieve the constitutional symptoms ascribed to the mere patchy excoriation; and, though papular eruptions do certainly now and then follow a severe gonorrhœa, such an occurrence is by no means common: nay, it may be deemed even an unusual sequela of that disease.

The length of this discussion obliges us to pass over Mr. Carmichael's observations on the distinct origin of chancre and gonorrhœa, as well as his extracts from the able pamphlet of Mr. EVANS: we hasten, therefore, to the consideration of Mr. Carmichael's doctrines.

The primary symptoms above mentioned, he continues, are liable to be followed by a papular eruption ending in desquamation: this has been the case in every instance in his practice during fifteen years, excepting in two. The constitutional symptoms ushering in this eruption are, more or less fever, with pain in the head and shoulders and larger joints, and sometimes pain in the chest, with dyspnœa; the eruption appearing on the forehead, chest, and back, and extending to the extremities. The fever continues to exist as long as fresh crops of eruption continue to appear; and the pains in the joints are more severe at night. The papulæ vary from a pale red to a crimson; some are pimples merely, whilst others are *almost* advanced to the pustular form. These spots do not come out all at once, but in succession, and are therefore met with in different stages of their progress,—some with acuminated tops, containing pus or lymph; others on their decline, consisting of exfoliations of the cuticle. In their latter stage their colour is coppery, and the desquamation of the cuticle gives them the appearance of scaliness, similar to the scaly eruption of syphilis. The distinction, our author tells us, is made by observing in the same person other spots in their papular or pustular form; and a further difference is, that, in this form of eruption, the copper-coloured scaly surface is more raised in its centre than in its circumference, which is directly the reverse in the scaly eruption of syphilis.

There is also another form of eruption, not so frequently met with,—that is, the papulæ are more minute and more clustered together. Of this form of disease sore-throat is a common accompaniment, but it is totally different from the excavated ulcer of the tonsils met with in syphilis ; there is considerable pain and difficulty of swallowing; and the entire fauces, particularly the back of the pharynx, exhibit an erithematous appearance, frequently with considerable swelling of the tonsils, which assume an irregular appearance, that is often mistaken for ulceration. (We may here remark, that true ulcerations most undoubtedly often attend this condition of the throat.) The cervical glands, continues our author, often swell and ulcerate in these diseases, especially when the eruption is on the decline: and this, he observes, has often been attributed to scrofula having been excited in the habit by the mercury employed. This opinion, however, he declares to be untenable, since the patient is equally prone to those swellings when mercury is not exhibited. This *fact* we are very much inclined to doubt; but, even were it not so, there can be no doubt that scrofula may be excited in a strumous habit, without the exhibition of mercury, by the mere debilitating effects of disease. The eruption, after having disappeared, is liable to return again and again at uncertain intervals, but always in a less degree, and with longer intervals, as the disease exhausts itself or yields to the powers of the constitution; but if mercury be employed before the disease has arrived at its latter stages, it becomes more obstinate and complicated than it would otherwise have been. Thus, if that medicine is exhibited on the first appearance of the eruption, and while fever is present, with severe pains in the joints, the patient is rendered much worse. We are extremely happy to add our testimony to the importance of this practical rule: it is, indeed, a golden one in this class of complaints. When the fever is overcome, the eruption will, in most instances, disappear under the use of mercury, and the pains will be alleviated, though not removed ; yet, when the mercurial irritation has ceased, a fresh crop of eruption will in general make its appearance, together with an increase of pains in the joints, and perhaps soreness of the throat.

From this last paragraph we dissent. That it may, and does, so happen, there can be no doubt ; but we believe that it only occurs in consequence of the mercurial irritation not having been kept up for a sufficient length of time: though it is undoubtedly true, as Mr. C. remarks, that the symptoms do recur even under the employment of mercury; and, wherever that happens, there can be no doubt that the advice he gives of resorting to the sarsaparilla, and abandoning the use of mercury, is most judicious.

If the advanced stage of this disease, but more especially if the eruptive stage has been superseded by the use of mercury, or if the eruption has been repelled by imprudent exposure to cold, inflammation of the iris of each eye is a common attendant. Mr. C. believes that this affection accompanies the papular eruption exclusively, but he does not positively affirm it; yet surely he is severe in his censure upon the author who controverts his position, and whose sole fault appears to have been that of calling our author a "speculative" writer. Mr. C. calls this a sneer. We are by no means convinced that it was meant as such; and he has not shown much forbearance in bestowing upon his opponent the title of a puny aspirant after fame, nor in condemning the publication of an octavo volume, which *he* considers as not containing one single fact of practical utility. Surely, such severity of remark was not called for in this instance: the road to fame is open to all, and he who fairly states his opinions may be wrong, but cannot be a just object of reproach or contempt.—But to return.

In many thousand cases of this form of disease, our author has not met with a single instance of decided nodes, or of affections of the deeper-seated parts; so that their absence may be considered as one of the characters of the papular disease.

We come now to *treatment.* That of the primary symptoms is very simple: it consists of perfect rest, and, if there be much inflammation, in enforcing a recumbent position. The diet is to be light, or strictly antiphlogistic. Cathartics and antimonial medicines are to be exhibited. These ulcers will heal under the use of any simple astringent washes, or mild ointments; but, if they continue long obstinate, mercury, in alterative doses, will certainly hasten the cure; but this our author attributes to the excitement of a new action of the system, and he uses it as he would do for the purpose of hastening the cure of chronic ulcers on the legs or elsewhere. Upon this point we are completely at issue with Mr. Carmichael: we do not hesitate to say, that if these sores, which are rebellious to common treatment, were under proper confinement, and treated with a judicious and temperate exhibition of mercury, continued until they are thoroughly healed, the subsequent constitutional symptoms, which he has so accurately and vividly described, will not ensue; and that we shall find, the earlier recourse is had to this remedy, (always provided that extensive inflammation and constitutional derangements do not contra-indicate its use,) the less chance there will be of the occurrence of any of these perplexing forms of disease, which we now meet with in every corner, since we have chosen to depart from the broad road of practice: and, upon Mr. C.'s own principle, we advocate this plan the more warmly, since he acknowledges that mercury will hasten the cure of these ulcers, and is also of opinion that, the sooner they are healed, the more likely is the constitution to escape contamination. (P. 101.) From this consideration, he advises the destruction of the surface of the ulcer by lunar caustic, and afterwards continues an application of a solution of the same substance.

We omit several pages devoted to the treatment of phymosis attended with high inflammatory action, simply observing, that the rules laid down must meet with the approbation of the judicious surgeon.

Mr. C. advocates the employment of acetic acid for the removal of warts, which frequently follow the healing of these, as well as of other ulcers of the genitals, and over which mercury has certainly no control.

Of the cure of the patchy excoriation much need not be said; any astringent application will answer the purpose. We know not who the persons may be who are asserted to subject their patients to a five or six weeks' course of mercury for this simple affection: we think that no reputable practitioner would make such a mistake; and we do not like the note which, at the bottom of page 109, appears to refer to this subject.

Of the treatment of gonorrhœa virulenta we shall say nothing, since our author does not differ in his opinions and practice from the majority of his professional brethren.

With respect to the treatment of the constitutional symptoms of the papular venereal disease, we are told they will yield, in every instance, to the powers of the constitution; but it will sometimes require several months to overcome the disease, which will appear again and again, until it has worn itself out. However, the cure may be hastened, when the disorder is on the decline, by the exhibition of alterative doses of mercury. The eruption being ushered in with fever, recourse at first is to be had to antimonials, bleeding, and the antiphlogistic regimen: after that stage is removed, antimonials are to be combined with sarsaparilla; but, if the pains and eruption continue to linger, the compound calomel pill, with decoction of the woods, is recommended; which has in no instance disappointed our author in removing this form of the disease. Such are Mr. Carmichael's doctrines with respect to this form of the venereal disease, and which he illustrates by several cases.

In reference to the inflammation of the iris, as a concomitant symptom, we find it asserted that it is often the result of the exhibition of mercury; that it is frequently the consequence of the disease; and that, though it is produced by mercury, it is equally curable by the same remedy: though we are told that Dr. THOMSON has cured many cases without its exhibition. However, Mr. C.'s practice is to throw in mercury as speedily as possible; not neglecting, at the same time, to put in practice both local and general blood-letting, blisters, the use of belladonna, and the antiphlogistic regimen.

In summing up this long chapter, our author trusts that he has adduced sufficient evidence to satisfy any reasonable mind that the same virus may produce the three primary affections described in the chapter; that they are all liable to be followed by the same train of constitutional ailments, "and that all the symptoms of this disease, both primary and constitutional, may be easily recognised and distinguished as to their external characters, by those who will endeavour to discriminate one disease from another. When all venereal complaints were treated nearly alike, such discrimination may not have been thought necessary for practical purposes; but, now that the baneful effects of this mal-practice is universally felt and acknowledged, the student, if he hopes for success, must learn to discriminate appearances, in order that he may judge of the true nature of the disease, and whether it is likely to be mild or malignant, brief or tedious, in its duration. And, finally, he may acquire a knowledge from those characters and appearances which will enable him to determine, with promptitude and decision, the mode of treatment best adapted for the case committed to his charge." (P. 138.)

In this paragraph, we cannot agree. That all venereal complaints should be treated exactly alike, we neither advocate nor propose; but that the baneful effects of such mal-practice is universally felt, we consider to be quite a gratuitous assumption: on the contrary, the mal-practice in venereal complaints we consider to be that now too generally adopted, viz.—leaving ulcers to simple dressings and mild applications,—suffering the constitution to be tainted, where the remedy to prevent it is within our reach,—refining upon refinement, until we have lost all chart and compass to steer our course by: and what has been the result? Such an abundance of constitutional complaints,—such a mixture of perplexing and anomalous cases, that the cure of the venereal disease appears to have retrograded a century.

[To be continued.]

Art. II.—*On Medical Education. Being a Review of "A Dialogue between the Author* (Dr. Paris) *and a Practitioner, who is about to direct the Medical Studies of his Son."**

We know not whether Dr. Paris has taken the idea of his present Preface from "La Nouvelle Helouise" of Rousseau, or Darwin's "Botanic Garden," but these are the only works we recollect to which a dialogue is prefixed, on a plan similar to that before us. However this may be, the design, and probably the effect also, in all such cases, is the same—to secure to the Preface a chance of being read; an advantage which, we suspect, does not always fall to the lot of introductory remarks.

A practitioner is supposed to come to London after an absence of nearly thirty years, (during twenty of which he has ceased to attend to the progress of medical science,) for the purpose of regulating the professional education of his son, a youth of nineteen, grounded in classical knowledge, and possessing some general ideas upon the subject of natural philosophy; but, "with regard to physic, with the exception, perhaps, of a little information on subjects of pharmacy, he is entirely ignorant."

Dr. Paris commences by alluding, in a general way, to the progress made in medical science during the last few years, and then proceeds more pointedly to censure the present system of teaching.

"*Author*. During the period you mention, general knowledge has made a rapid stride; and it would have been 'passing strange' had not medical science participated in the advancement; but I am by no means satisfied that our system of *teaching* has been improved. It is true that amongst our metropolitan lecturers may be ranked some of the first philosophers of the age; but there are many competitors, some of whom, to maintain their ground, have introduced a system of '*grinding*,' or '*cramming*,' as it is technically called in our universities, which allures pupils, from the assistance it affords them in passing an examination at the College of Surgeons, or at Apothecaries' Hall; but is, in my humble judgment, ill calculated to impart solid information.

"*Practitioner.*—This remark is not new to me. I have often been astonished at the harlequinade dexterity with which a college licence has transformed the new student into the sapient practitioner, and the humble scholar into the dogmatical lecturer."

We agree to a certain extent in these remarks; but must protest against the sweeping condemnation which an unqualified assent would imply. Had the censure been limited to the substitution of *grinding* for the more regular and legitimate method of unfolding the principles of science in a systematic course of

* Prefixed to Dr. Paris's *Elements of Medical Chemistry*.

lectures, we should have concurred with Dr. Paris to the full extent of his expressions. But, on the other hand, we are decidedly of opinion that medical lecturers ought not to resemble in any degree the fashionable clergymen, so well described by Cowper, who

> —— "pronounce a text;
> Cry, hem; and, reading what they never wrote
> Just *fifty* minutes, huddle up their work,
> And with a well-bred whisper close the scene."

There is a medium in this as in every thing else, and we are satisfied, from considerable intercourse with pupils, that a short time may be most beneficially spent, after each lecture, in giving to the student an opportunity of seeking for further illustration on any part of the subject he may not have fully comprehended, and even of ascertaining, by categorical inquiries, how far the views of the teacher have been understood and appreciated by his hearers. This, it is true, is an additional labour to the lecturer, and by some may be regarded as *infra dignitatem;* but he who professes to instruct should consider nothing as beneath him which facilitates the acquisition of that knowledge he undertakes to impart. To the pupil, the advantage is great and unequivocal: it proves an additional spur to his attention, —it makes him acquainted with his own deficiencies, and enables the teacher to point out to him the sources of information best calculated to remove them. It may, perhaps, be thought that the strictures of Dr. Paris do not apply to the case we have described, and consequently that our remarks are uncalled for; but, where a great difference exists, a broad line of demarcation should be drawn,—and this is our object. Against that system which would *substitute* grinding for the more regular method of teaching formerly adopted, we protest as strongly as Dr. Paris; and, like him, we are aware of its existence. We know that there are some who even profess to fit pupils for passing their examinations,—and who, if they succeed in this, certainly fit them for nothing else. The knowledge of medicine thus acquired consists not in the power of detecting disease at the bedside of the patient, but of assigning it a learned name in some System of Nosology; and their anatomical skill will not be in the readiness with which they can use the knife, but in their being able to give the origin and insertion of certain muscles, by a great and *temporary* exertion of the memory; an intellectual pitch to which they are wound up like any other pieces of machinery, and which just lasts long enough to carry them through the ordeal of Lincoln's-inn Fields. But, while we object to this system, we repeat our conviction of the advantages to be derived from that kind of discipline to which we have above alluded, and which, if it allures pupils, does so not "from the assistance it affords them in passing an examination at the College of Surgeons or Apothecaries' Hall," but from the facility which it gives to the great object of all their studies, —the acquisition of professional knowledge.

The dialogue next turns upon the various circumstances which contribute to the success of a professional man, and the view taken of the subject appears to us to be correct. The author remarks, that, after the extraordinary success of his friend's townsman, he concludes that nothing short of the rank of a London physician will bound his expectations; to which the other replies—

"*Practitioner.* The case, my dear sir, to which you allude, must tend to extinguish rather than encourage such ambition. I never dwell upon the extraordinary success of that man, but the Persian fable immediately intrudes itself upon my recollection. 'A drop of water fell out of a cloud into the sea, and finding itself lost in such an immensity of fluid matter, broke out into the following reflection: "Alas! what an insignificant creature am I in this prodigious ocean of waters; my existence is of no concern to the universe; I am reduced to a kind of nothing, and am the least of the works of God." It so happened that an oyster, which lay in the neighbourhood of this drop, chanced to gape and swallow it up in the midst of this its humble soliloquy. The drop,' says the fable, 'hardened in the shell, until by degrees it was ripened into a pearl, which, falling into the hands of a diver, after a long series of adventures, is at present that famous pearl which is fixed on the top of the Persian diadem.'

"*Author.* From which I am, of course, to conclude, that you consider the worldly success of a physician to be alone dependent upon accidental circumstances which no wisdom can foresee, nor any prudence control.

"*Practitioner.* Such, I believe, was the opinion of no less a person than Dr. Samuel Johnson, whose judgment you will scarcely venture to question.

"*Author.* I am ready to admit that 'Victory is not always to the strong;' and I am well acquainted with instances in which superiority of success has been united with inferiority of pretensions: but, as Dr. Young has very justly remarked, whatever may be the accidental irregularities inseparable from the operation of moral causes, it must be admitted that every man's chance of success in his profession will be in some measure proportionate to his merits and his talents;—in the lottery of physic, as in all other lotteries, the chance of a man who holds ten tickets must be decidedly better than one who is possessed but of five. The same argument will apply to the courteous address and behaviour of a practitioner: we have seen the most unpolished, and even indecorous, manners distinguish the most successful, but are we, on that account, to argue that urbanity and kindness are of no avail? Dr. Radcliffe told Dr. Mead, as a great secret, that the true way to succeed in physic was to use every body ill; but Dr. Mead used nobody ill, and succeeded better than Dr. Radcliffe." (P. iii.—v.)

There is doubtless much truth in these observations: the advice of Dr. Radcliffe was decidedly wrong, and we have some striking living instances of the advantages of prepossessing manners in the medical profession. Perhaps, in order to obtain ultimate success, it is not desirable that the eyes of the public should be too soon fixed upon any individual, before he has had time to establish some reputation among his professional brethren, so as to enable him to bear up against any of those contingencies to which all practitioners are liable. The first shock a man meets with, who has no character at his back,—he falls; and, so far as our own limited experience serves us, little is gained by what is commonly called, attempting "to take the town by storm."

With a view to the future success of our student, the "Author" advises that, if intended for a physician, he should forthwith be entered at Oxford or Cambridge; and, on the Father objecting that these are not medical schools, he continues—

"*Author.* It is most extraordinary to me that this senseless cry should be so long continued. Is technical knowledge all that is required for the accomplished physician? Do not the liberal pursuits, which are so successfully cultivated in those seats of learning, contribute to the elevation of the understanding; to say nothing of the gentlemanly manners and feelings which are thus acquired by intercourse with the most exalted characters of the age? Why is the rank of the regular physician so much higher in this than in any other country? Because he must receive his education in the same school as that in which the nobles and statesmen of the land are instructed." (P. viii.)

There can be no question as to the soundness of this advice, —at least, if the son be to practise as a physician in London; and we think Dr. Paris has given a fair view of the advantages to be therefrom derived. He claims not superiority of medical education; but it is no small advantage that he who is to practise among the higher classes of society, should have his manners and feelings cast in the same mould as theirs: added to which, he frequently forms friendships with men who afterwards take a lead in society, and by whom he is supported and advanced in his professional career. There is, however, yet another advantage in going to either of the English universities: we mean that it enables him to become a Fellow of the Royal College of Physicians in London, by which he secures the undisputed acknowledgment of holding the highest rank in his profession, and shares in the common benefits resulting from that *esprit de corps* among the members, which, however they who are less fortunately situated may regret, they yet can scarcely blame. The Licentiates, if they were alive to their own interests, would do the same: but they have no common bond of union, no "local habitation;" and, so long as the present constitution of medical politics endures, a Fellow of the College of Physicians has, *cæteris paribus*, a better chance of success than a Licentiate.

With regard to the succession in which the different branches ought to be studied, Dr. Paris says—

"*Author*. I should recommend Anatomy as the first object of his pursuit; a knowledge of this branch cannot be too early attained: enter him, therefore, at once with some good teacher, and, after he has attended one course of lectures, let him commence dissection; at the same time he should be admitted as a pupil at some hospital, in order that he may become practically acquainted with the history and treatment of disease; his attendance also at lectures on the theory and practice of Physic will be necessary. Such a course of study will be amply sufficient for his first season. In the second year of his residence in London, he may attend to lectures on Surgery and Midwifery, and on Chemistry and the Materia Medica. The third year may be advantageously spent in Edinburgh, which is unquestionably one of the first medical schools in the world, greatly inferior,* however, to London, with regard to anatomy; and on that account the first year of study will be better spent in London, and he will be thus enabled to receive a greater profit from the excellent clinical and practical lectures which are annually delivered in Edinburgh. During the summer recesses, he will do well to cultivate a knowledge of Botany, and to enter upon a course of medical reading; he may, at the same time, amuse himself with the repetition of the various chemical experiments which he had witnessed during his attendance at lectures." (P. v. vi.)

We are not satisfied that the order here suggested is the best that can be followed: we would be disposed to recommend anatomy, chemistry, and materia medica, as the rudiments of medical science, to be assiduously and exclusively studied during the first year; and the theory and practice of medicine and surgery during the second, when the student would be much better able to appreciate them. We entirely agree with what follows regarding the general and indiscriminate system of taking notes: it is better for the student to carry the lecture in his head than in his pocket.

"*Author*. No pupil should make the attempt during a first course; for, in the first instance, the subjects will be so novel as to require the entire devotion of the student's mind; and, if he attempt to reduce any part to writing, he will lose the thread of the discourse, and be unable to follow and comprehend the lecturer. When, however, he retires to his chamber after the lecture, much advantage will arise by his making memoranda of such facts or opinions as may have appeared to him the most striking. In the second course, the practice of taking notes is of decided utility; but the use of short-hand on these occasions is in every way to be reprobated. It converts, says Dr. Young, the writer

---

* "In consequence of the difficulty of obtaining recent subjects. This embarrassment, however, now threatens the London schools."

into a mere machine; it employs him in copying words, instead of digesting and compressing thoughts; and, unless he has two or three more hours to bestow on the same subject after the lecture, which very few lectures are worth, his manuscript remains in a form almost as inconvenient for reference as if it were written in an unknown language." (P. vii.)

The rest of the dialogue is entirely devoted to illustrations of the advantages conferred on medicine by chemistry; but into these it would be foreign to our present purpose to enter, as we took up the article only with reference to the author's remarks on education. We have differed from him on some points, and therefore would remind him (to borrow one of his own quotations,) "Licet omnibus, licet etiam mihi, dignitatem Artis Medicæ tueri; potestas modo veniendi in publicum sit, dicendi periculum non recuso."

---

### DIVISION II.

### FOREIGN.

ART. III.—*Manuel d'Anatomie Générale, Descriptive et Pathologique*; par J. F. MECKEL, Professeur d'Anatomie à l'Université de Halle. *Traduit de l'Allemand, et augmenté des Faits nouveaux dont la Science s'est enrichie jusqu'à ce jour;* par A. J. L. JOURDAN, Membre des Académies Royales de Médecine de Paris, &c. et G. BRESCHET, Professeur agrégè en Exercice, Chef des travaux Anatomiques de la Faculté de Médecine de Paris, &c. Tome troisième.—Paris: Baillière, 1825.

[Continued from page 340.]

WE resume our analysis of M. MECKEL's work, hoping to include in the compass of this and one more article, the pathology of the Skin, of the Glands, and his chapter on Accidental Formations, which will finish the first volume of the work; and, in fact, the only one available for the purposes of analysis. We commence with the

*Pathology of the Muscular System.*

The substance of the muscles is not regenerated, and wounds of these parts, unattended with loss of substance, are cured in the same manner as those which are accompanied by loss of substance: indeed, they resemble them very closely, in consequence of the distance produced between the divided parts by the contraction of the muscular fibres. In either case, the vacant space presents the appearance of a depression, around which the lips of the wound are a little tumefied: this is filled soon with a reddish, soft, and gelatinous vascular mass, which afterwards becomes of a yellowish white, hard, and coriaceous, and which is at all times insensible to the action of every kind of stimulus. Occasionally, many months after the wound, certain irregular fibres are met with; but these have no analogy to the muscular substance; and, if a muscle be completely divided throughout its whole thickness, the two halves are so completely separated, that an irritation applied to the one causes no corresponding contraction in the other. But, in spite of this separation, they are equally nourished, and they do not dwindle, as a nerve under the same circumstances would do; which depends, no doubt, upon the muscles not forming, like the nerves, an organic system united together in all its parts. A muscle having a transverse wound, which has become cicatrised, is really converted into a digastric muscle, resembling one, the continuity of which is interrupted by tendon.

Muscles deviate in many respects from their healthy state, both as regards their form and chemical composition, and their action. Our author only examines into the two former of these conditions. Among the errors of conformation may be mentioned an unnatural number: this is almost always a congenital defect. Sometimes all the muscles are wanting, although the other parts are formed; but this can only happen when the development of the whole body is very imperfect, particularly when the upper half of the body is unformed, and a gelatinous mass only occupies its place. Nevertheless it has happened that, in these instances, the muscles have been overlooked in consequence of their white colour, as well as of the presence of a great quantity of fluid under the skin. It is less uncommon to find certain muscles wanting, either altogether or in part; such as the palmaris brevis, the plantaris, the pyramidalis, and some fasciculi of the flexors of the fingers or toes. Supernumerary muscles are rarely met with: nevertheless, some examples are pointed out by M. Meckel; of which, the great dorsal, the pectoral, and the extensors of the toes, are the most remarkable.

The muscles are sometimes met with larger or smaller than natural, but this deviation is seldom congenital; most commonly it arises from some accidental circumstance. The diminished size is generally the result of want of exercise, and compression will often destroy them entirely; whereas their extraordinary power is generally the result of great exercise. This only becomes a disease when the muscle (the heart, for example,) acts with such excessive vigour as to disturb the functions of health. There are also some examples of a primitive defect in the attachments of muscles, which, by not attaining their natural insertions, become powerless, or even act in a manner contrary to the direction which nature meant them to possess.

Anomalies in the connexion of muscles are commonly accidental; they are either confined to the muscles themselves, or extend to their relations to other parts. The phenomena attending wounds of these parts have already been explained. It is not unusual to find, when the external parts are not injured in the smallest degree, in the dead body, entire muscles, or particular fasciculi, torn, with an effusion of blood at the torn part. These ruptures are probably occasioned by spasmodic contractions occurring in the last moments of life. However, the loss of substance is sometimes consecutive to the effusion of blood. Continued pressure may also destroy a part of a muscle, and in this manner separate the connexions which existed between it and other muscles.

With regard to the displacement of muscles in relation to the neighbouring parts, they are generally the result of adhesions contracted by previous inflammation. Inflammation may also produce the adhesion of muscular fasciculi to each other, causing a greater or less degree of rigidity. Amongst the alterations of the texture of muscles, their different degrees of cohesion may be mentioned: they are sometimes exceedingly flaccid, and easily torn; on the contrary, they are met with more elastic and firmer than ordinary. The first condition is met with after diseases of debility; the second exists independently of any other morbid state, and is met with especially in the hollow muscles, such as the bladder, but more particularly the heart. The colour of muscles is sometimes variable, although there is no change in the texture; but occasionally they are met with together, as in palsy or dropsy. In rheumatism, which attacks principally the muscular sheaths, a gelatinous fluid is generally found effused between it and the surface of the muscle. Paleness and softness of the muscles mark the progress of an alteration of texture, which is not very commonly met with, and which sometimes follows their inaction. This is the conversion of the muscular substance into fat, whether it loses its texture or appears simply like cellular substance filled with fat. In this case the muscle becomes smaller than in a natural state. Fatty tumors are not often formed among the muscles, and ossific formations, or tuberculous, schirrous, or fungous growths, are still less so.

Hydatids are occasionally found in the mucous tissue which unite the muscular fibres. This has been observed as well in those muscles belonging to organic as to animal life, and especially in the heart. An accidental formation of muscle never takes place: it has been pretended that the serous membranes, and the bones themselves, have been converted into muscle, and it has been declared to have been found in the ovaria; but there is no doubt that in these instances the distinctive characters of muscle have been overlooked.

*Of the diseased States of the Serous System.*

The serous membranes deviate from their natural state in a very remarkable manner, as well in their configuration as in their texture. The principal defects of primitive conformation are—1, the absence of a portion of these membranes, such as the pericardium, the pleura, or the peritoneum; 2, an unnatural communication between different portions of this membrane,—for example, between the tunica vaginalis and the peritoneum. But other primitive errors of conformation are to be met with; such, for instance, is the existence of a serous sac in the interior of a natural cavity, with which it communicates by an opening of a greater or less size, which contains a portion of the viscera, and separates them from the rest. This is only met with in the peritoneum. The fact is remarkable as an example of an unnatural repetition of a natural formation. The serous membranes are also liable to errors of consecutive formation, as is the case in hernia. In general, when this is the case, a portion of the serous membrane detaches itself from the parietes of the cavity to which it is adherent, and passes through an opening in these parietes, either naturally large or widened by the action of some external cause, and thus forms an hernial sac, into which some of the viscera escape. It seldom happens that this sac is torn, or that the viscera escape from the cavity without being preceded by it, so that the hernia is without a sac. This event only takes place from the effect of considerable violence, or where the hernia takes place at a certain point; for example, at the upper part of the peritoneum.

The serous membranes are frequently distended to an enormous degree by the fluids they exhale, constituting dropsy. Ordinarily the fluid of dropsical subjects may be considered as the serum of the blood, which has lost from two-thirds to four-fifths of its albumen; though sometimes the albumen is increased in quantity.

The other alterations which take place in the serous membranes are the consequences of previous morbid states, especially of inflammation. This affection of the serous membranes has a great tendency to terminate in effusion into their interior, the result of which is a thickening of their substance; or by exudations on their surface, which produce an adhesion of the corresponding surfaces of the external and internal sac, without their having been previously injured by suppuration: these adhesions differ greatly as to extent, solidity, and number.

It is extremely probable that these adhesions are never primitive, although Bichat affirms the contrary in regard to certain perfectly organised ligaments, which are met with between the external and internal surfaces of the pleura, and which are manifestly composed of two layers placed one against the other. Tioch, however, agrees with Bichat in regard to certain analogous formations between the heart and pericardium, on account of their resemblance to those found in the hearts of several reptiles in a state of health. But it is certain that the most perfect organisation of these productions is not sufficient to justify this opinion, because other parts, still more perfectly organised, as bones, teeth, and even entire serous membranes, are developed as the consequence of a vital action, between which and inflammation there is no essential difference. Those alterations of texture in serous membranes, which are characterised by their thickening, do not present every where the same appearances: thus, in the internal sac of the pericardium, they form large, smooth patches; and in the peritoneum, a number of small, hard, round elevations, which bear a close resemblance to a miliary eruption.

The serous system has a great tendency to ossification: sometimes the substance of the membrane becomes ossified; sometimes smooth round bodies are formed upon its surface, variable both in number and extent, which are occasionally loosely attached, at others become detached from it, and float in its cavity. Of all the portions of the serous system, that part of the peritoneum lining the spleen has the greatest tendency to ossification; the tunica vaginalis next; between the others, except the arachnoid, in which ossific formation seldom takes place, there is but little difference. Almost all these accidental ossifications take place in patches, which acquire, especially in the spleen, a considerable size, so as occasionally to usurp the place of the membrane itself. It is very common to find bony concretions formed in the substance of the synovial membranes, as well as in the bursæ mucosæ. Nevertheless, these concretions are not exclusively met with in them; for they are to be found in what are strictly called the serous membranes, the tunica vaginalis testis, peritoneum, the pleura, and arachnoid.

Generally speaking, these concretions arise in the above manner, but occasionally they are originally free from any attachment, taking their origin either from blood or some other effused fluid, in consequence of external violence; though even in this case there is reason to suppose that some connexion has been formed between the extravasated fluid and the synovial membrane, before the formation of bone commences. The formation of these concretions in serous membranes which have no connexion with bone, proves, however, that the vicinity of bone has no influence (as Hunter thought) upon the conversion of the effused fluid into ossific matter.

Independently of these anomalies, which are sufficiently common, others are met with, but more rarely: such as, for example, the development of an immense number of loose prolongations, soft, and some lines in length, from the internal surface of the synovial membrane of the knee; though this, perhaps, might have been but the first step of that process by which the ossific concretions are formed.

The serous tissue has, of all others, the greatest tendency to be multiplied in an unnatural degree in the human body. Those serous membranes of accidental formation become even the basis of other unhealthy formations: such are the cysts of encysted tumors, which present all the essential characters of serous membranes. They always consist of sacs closed on all sides, smooth internally, and covered with asperities on the outside; they are produced by the mucous tissue; have few blood-vessels, and fulfil the same functions as the healthy serous membranes, although the substances contained within them are not always of the same nature with the serous fluid, nor indeed always fluid. It is probable that these cysts are not formed, as is generally thought, in a mechanical manner, by the compressive action of an effusion into the cellular substance. Bichat had combated this theory by reminding us that these cysts bore the strongest resemblance to the serous membranes; that secretion continued to go on from their internal surface, whereas compression rendered them impermeable; that the cellular membrane is not diminished in the neighbouring parts; and that, should this hypothesis be admitted, it would imply that the secreted fluid was pre-existent to the secreting organ. He thinks that these parts are formed as all others are in the mucous tissue, and that secretion does not commence until their structure is completely developed. Nevertheless, it cannot be proved that the formation of a cyst has not been preceded by extravasation into the mucous tissue, though the cyst is not developed in consequence of the fluid compressing that tissue, but is formed at its own expence, because it possesses the property of becoming organised. This theory appears extremely probable, as well on account of the analogy of structure and function which exist between the mucous and serous systems, as on account of the pathological phenomena in the midst of which these cysts are produced. In fact, it is by no means uncommon to find, either in the cavity of the natural serous membranes, or in cysts of accidental formation, an immense number of small cysts perfectly detached, without any trace of previous adhesion, and filled with a serous fluid, most commonly limpid. These little cysts, called hydatids, are surrounded by an analogous fluid. It is evident that they could only be formed at the expense of a fluid effused in the cavity of a serous membrane, in consequence of its separation into two parts, the one solid, the other liquid. According as this fluid is either effused into the mucous tissue or into a serous membrane, the cyst to which it gives birth becomes united to the neighbouring parts by means of the surrounding cellular membrane, and receives blood-vessels, or remains free from all adhesions. The serous membranes have more tendency than any other organ to produce these different kinds of cysts; and, even when they appear to have been developed in the substance of the viscera, (the liver, for example, which is found sometimes entirely destroyed,) it is probable that its formation commences in that portion of the peritoneum lining it; for they are always found applied to the surface, and upon some point of its circumference.

### *On the Pathology of the Skin.*

The skin possesses the faculty of regeneration in a very high degree. All its parts are reproduced after destruction, from whatever cause; but they are not reproduced with characters perfectly resembling those which it possesses in a healthy state. The dermis is less elastic, and adheres more closely to the subjacent mucous tissue than in its natural condition: it, indeed, makes but one substance with that tissue, and cannot be separated from it. Like all regenerated parts, it has less durability and less activity than the healthy skin; which explains the facility with which cicatrices, even of long standing, are ruptured, and the ease with which the integument formed on the surface of cutaneous ulcers is entirely destroyed. At first the new skin is very thin, delicate, and soft, more vascular, and consequently redder, than the sound skin; but by degrees it becomes less vascular and whiter, as well as more solid; it acquires almost the properties of ligament; at the same time it has a brilliant, shining aspect, which arises doubtless from the want of papillæ and hairs, as well as from its greater tension, and its more intimate adhesion to the subjacent mucous tissue. Its sensibility is also less than that of the healthy skin, and it probably receives fewer nerves. These phenomena are, however, only observable where the skin has been entirely destroyed; for, where it has only met with a superficial lesion, all traces of difference are lost in a greater or less space of time. The regenerated dermis is covered with a rete mucosum and epidermis; but the reproduction of these latter only takes place by slow degrees, the first layers which are formed always fall off.

That which is reproduced with most difficulty is the colour of the rete mucosum, and which sometimes is never restored. Bichat declares that the colour is never reproduced, and that the scars are equally white in all people; but that is not quite the case, since the scars of the small-pox are black in the Negro; and so are all those which succeed the solutions of continuity in the common integument of this race of people,—nay, sometimes they are of a deeper shade than the rest of the skin.

The nails are not only reproduced from the spot where they originally grow, but they have also been found to arise from the extremity of the second phalanx, after the loss of the third. Hair does not grow again where the dermis has been entirely destroyed; but, when they fall off after illness, they are always regenerated in a greater or less degree.

Diseases of the skin extend to all the layers of which it is composed, or are restricted to one only; and the same remark applies to the errors of conformation, and to the alterations of texture of this organ. The primitive defects of the skin are—1st, its absence: this may be either total or partial, either with regard to the body, or to the parts of which the skin is composed; 2d, an excess: as when a number of round or oblong excrescences are found upon different parts of the body, almost always accompanied by a defective development of this organ at some particular points. With respect to the different parts of the cutaneous system, the most striking example of the excess of any portion is the extraordinary length of the hair, in parts where it is usually very short: this is almost always found allied to a very dark colour of the skin, and coincident with the formation of fat in the part. The above errors of conformation may be secondary, as well as original.

The epidermis, the hair, and nails, are sometimes killed by diseases of the skin, or in consequence of other morbid conditions producing great debility of the vital powers; they then separate from the body. The epidermis is always reproduced; but this is not always the case with the hair and nails. The whiteness of the hair is also the result of imperfect nutrition, and is really connected with the rapid or gradual death of its internal substance. The skin, and its different parts, are also susceptible of enlargement or increase. The dermis may become thickened, and the papillæ elongated. Warts are instances of unusual increase in some points of the dermis; as corns are of a thickening of the epidermis. An unusual development of the epidermis constitutes the essence of icthyosis. There is considerable affinity between these different states and the excessive growth of the hair in the plica-polonica, and which forms the link of connexion between errors of conformation and alterations of texture.

Alterations of texture are very frequent in the skin. In the first place may be remarked the want of colour in the rete mucosum, constituting *leucosis*, which is most commonly congenital, though sometimes taking place in the course of life. Independently of different degrees of inflammation, to which different names are given, depending upon the part of the skin attacked. The cellular organ is subject to a great variety of affections peculiar to itself, which are called *exanthemata*, the history of which belongs to pathology. In general, in these diseases the skin bears some analogy to the mucous membranes; inasmuch as it receives more blood-vessels, becomes softer, it likewise affords fluid secretions, and the epidermis is almost always detached. As to the exanthemata themselves, their form is usually circular; they are attended with the usual characters of inflammation; and their result is the formation, in most instances, of a particular fluid. Chronic exanthemata have their seat generally in the substance of the dermis, whilst those whose progress is more rapid originate in the external surface of this membrane, and in the vascular tissue. Other unhealthy formations are developed primitively, in the subcutaneous cellular membrane,—such as wens, schirrus, cancer, and fungus hæmatodes, which nevertheless extend sooner or later to the skin itself.

We do not consider our author's observations relative to the accidental formation of the hair, nails, and corns, of sufficient importance to lay before our readers; and therefore proceed to consider his views of the *diseased conditions of the mucous membranes*, or, as he calls it, the *internal cutaneous system;* which he begins as follows:—

We are not in possession of any facts to enable us to decide whether the mucous membranes are capable of being reproduced when destroyed, or if, in those cases in which this has appeared to be the case, there has been merely a re-union of parts which have remained entire. The mucous membranes are subject to a great variety of anomalies; their errors of conformation, those especially which are congenital, coincide almost always with the analogous condition of the organs which are lined by them,—such as division, prolongation in the form of a *cul de sac*, retractions, inversions, &c. Those unnatural prolongations of the mucous membrane only form the link of connexion between errors of conformation and alteration of texture, since they sometimes consist only of simple prolongations, such as the valvulæ in the intestinal canal; but much oftener consisting of excrescences and new formations, the texture of which differs more or less from the healthy structure of this membrane. It may be remarked, that such deviations are generally found towards the extremities of this system, and near to its union with the external skin,—as in the nasal fossa, the cavity of the mouth, the pharynx, rectum, uterus, and vagina. These excrescences are called polypi; they adhere to the internal surface of the mucous membrane by a pedicle more or less short, broad or narrow, and float in the cavity which the membrane usually forms. Their structure is not always exactly alike; they are generally formed of a homogeneous substance, but sometimes fibres are found perpendicular to the surface which supports them. Their consistence also varies greatly; they are sometimes hard, at others mucous and soft. They occasionally receive a great number of vessels; in others, there are none distinguishable. They often occasion much trouble by their size and pressure, and injure the health by bleeding violently, either from their surface or from vessels ruptured in their substance. They now and then inflame and suppurate. The place from whence they have grown usually retains a strong tendency to reproduce them, after they have been extirpated.

Schirrus and cancer are equally the peculiar attribute of the mucous membranes and the glandular system, which may be considered as the result of the development of these membranes. These diseased productions are met with also at some particular points, and which are, generally speaking, the same as those on which polypi are met with. The genital organs of the female and the rectum are, however, the parts most commonly affected. They are also produced in other parts where polypi rarely appear; such as the stomach, especially where it joins the small intestines. This disease has its seat incontestibly in the mucous cryptæ, and owes its origin to the frequent irritation of these parts. It considerably diminishes the capacity of the organ, in consequence of the thickening which is its common result.

The mucous membranes rarely become ossified, or, at least, ossific matter forms only on their posterior surface; but it frequently happens that large fatty wens, of a round form, are generated in many places, among which the œsophagus and the small intestines may be named. Vicq d'Azyr and Munro have both denied this; having, in all probability, confounded them with other tumors of a different nature. The general condition which leads to all these anomalies is the exaltation of the nutritive faculty, *inflammation;* which, however, often attacks the mucous membranes without giving rise to such consequence. One of the most frequent results of this inflammation, especially when it has lasted some time, is a thickening of the membrane. Ulcerations also are not uncommon. But mucous membranes are capable of suppurating without ulceration; which, no doubt, depends upon the strong resemblance existing between their natural secretion and pus. The surface of inflamed mucous membranes secretes a quantity of coagulable substance, forming cylinders, either hollow or solid, as is observed in the croup. Nevertheless, it is very uncommon to find this class of membranes contracting adhesion after exudations of this kind; but they often unite in consequence of ulceration, particularly in those places where neither motion nor the passage of foreign matters afford an obstacle to such an union. Do the mucous membranes take any share in the formation of the exanthemata, which are so common in the external cutaneous system, and which show themselves under such varied forms? There is no question in pathology on which opinions are so much divided. There is no doubt that the exanthemata often appear in that portion of the mucous membranes near their junction with the external skin, when this last is itself attacked. But do the exanthemata assume the same form as in the skin? The great difference which exists between the texture of these two parts, authorises us to believe that they differ considerably; and experience teaches us that, in many of these affections, the small-pox for instance, all the mucous membranes are in a state of high inflammation; but they do not exhibit the slightest trace of pustules, although the skin is covered by them. Nevertheless, some observations, particularly those of WRISBURG and BLANC, incontestibly prove, in defiance of the contrary opinions of some very eminent physicians, that, when variolous pustules exist on the skin, they are also occasionally formed on the surface of the mucous membranes, particularly in the air-passages and in the intestinal canal; and that there is very little apparent difference between them.

Mucous membranes are produced under a great number of circumstances, most generally as a consequence of inflammation terminating in suppuration. Every surface that suppurates may be compared to an imperfect mucous membrane. At the termination of an inflammation, the cellular membrane, filled with the coagulable part of the blood which is effused into its substance, is transformed into a soft and whitish membrane, which soon acquires the property of secreting a peculiar fluid called *pus*. The great analogy between this and mucus is demonstrated by the insufficiency of our tests to distinguish between them. This membrane, which is intimately united to the subjacent cellular tissue, soon receives a great number of vessels: its surface, at first smooth, becomes unequal; a number of little tubercles arise, formed of vessels and cellular substance, which are called *granulations*. In this state it continues to secrete pus, until the vascularity diminishes and the granulations subside; and instead of them a substance, analogous to the healthy membrane which existed previously, is formed. Mucous membranes, then, fall into suppuration much more readily than other parts; and, what is more remarkable, they are capable of producing pus without any previous breach of surface. Accidental cysts have often also a great resemblance to the mucous membranes, as well with respect to their structure as to the nature of the fluid contained within them; and Bichat has certainly gone too far in referring them all to the class of serous membranes. In the ovaries and the uterus little cysts have more than once been found, which resembled the mucous more than the serous membranes. There is also, according to M. Meckel's belief, generally speaking, an exact relation between their structure and the nature of the fluid they contain; for those cysts whose contents are serous resemble very much the serous membranes, while those filled with a thicker matter, either mucilaginous or purulent, are more similar to the mucous class.*

[To be continued.]

### MEDICAL AND PHYSICAL INTELLIGENCE.

* We have given M. MECKEL's opinions throughout without observation or comment, only omitting a few passages which were not of primary importance. There are many points in which we differ from the learned author; but we have not chosen to extend these articles by mixing up with them any controversial matter of our own.—EDITORS.

